# Supplementary figures and images for: MyosinA is a druggable target in the widespread protozoan parasite Toxoplasma gondii
Source: PLoS Biol. 2023 May 8;21(5):e3002110. doi: 10.1371/journal.pbio.3002110 (PMC10185354; doi:10.1371/journal.pbio.3002110)

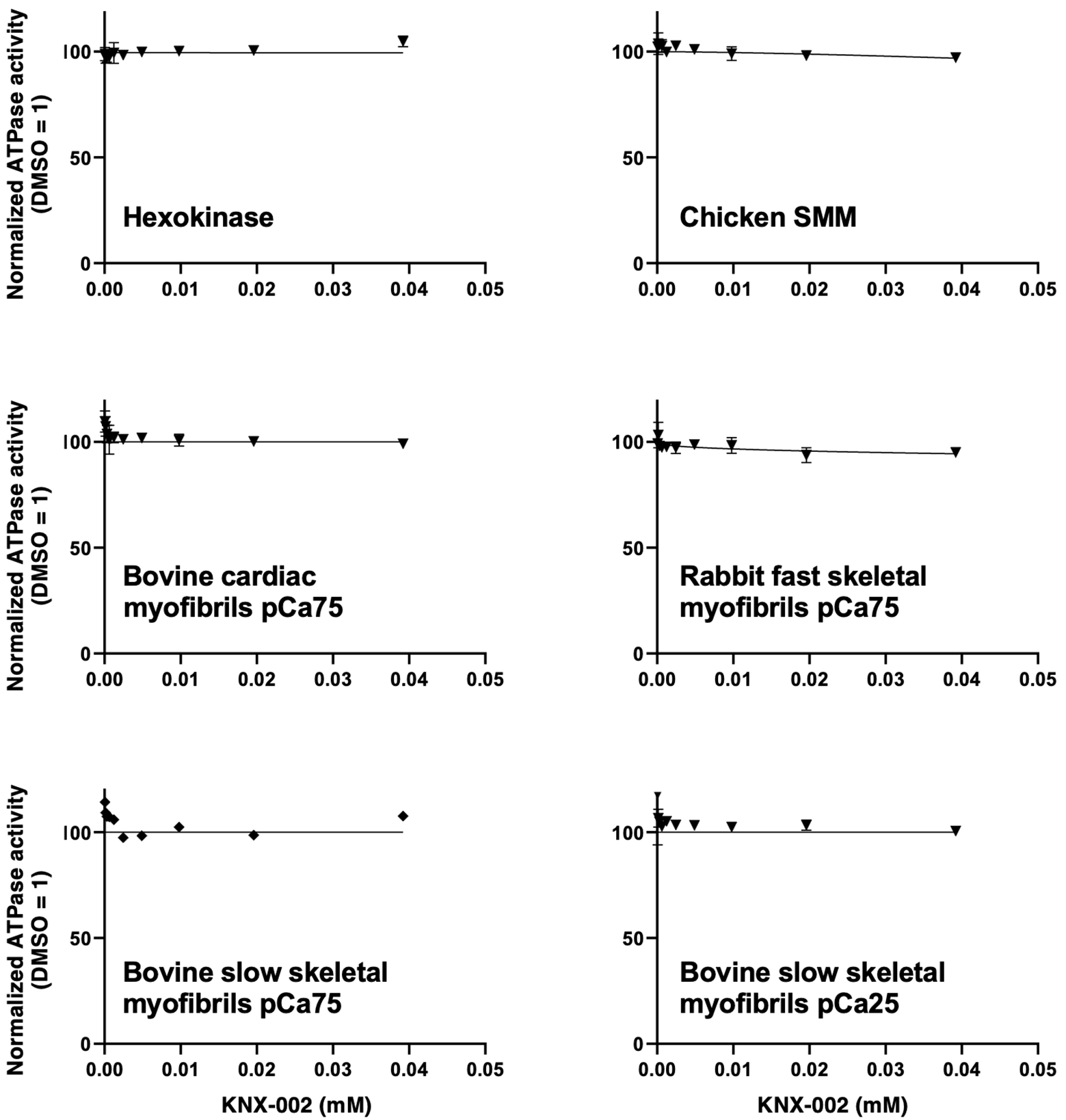

Supplement: S1 Fig — Dose-response curves showing the effect of KNX-002 on the actin-activated ATPase activity of various vertebrate myosins. SMM = smooth muscle myosin; pCa25 and pCa75 refer to assays done at 25% and 75% calcium activation, respectively. The measurements underlying the data plotted in this figure can be found in S1 Data. (TIF) [file pbio.3002110.s001.tif]

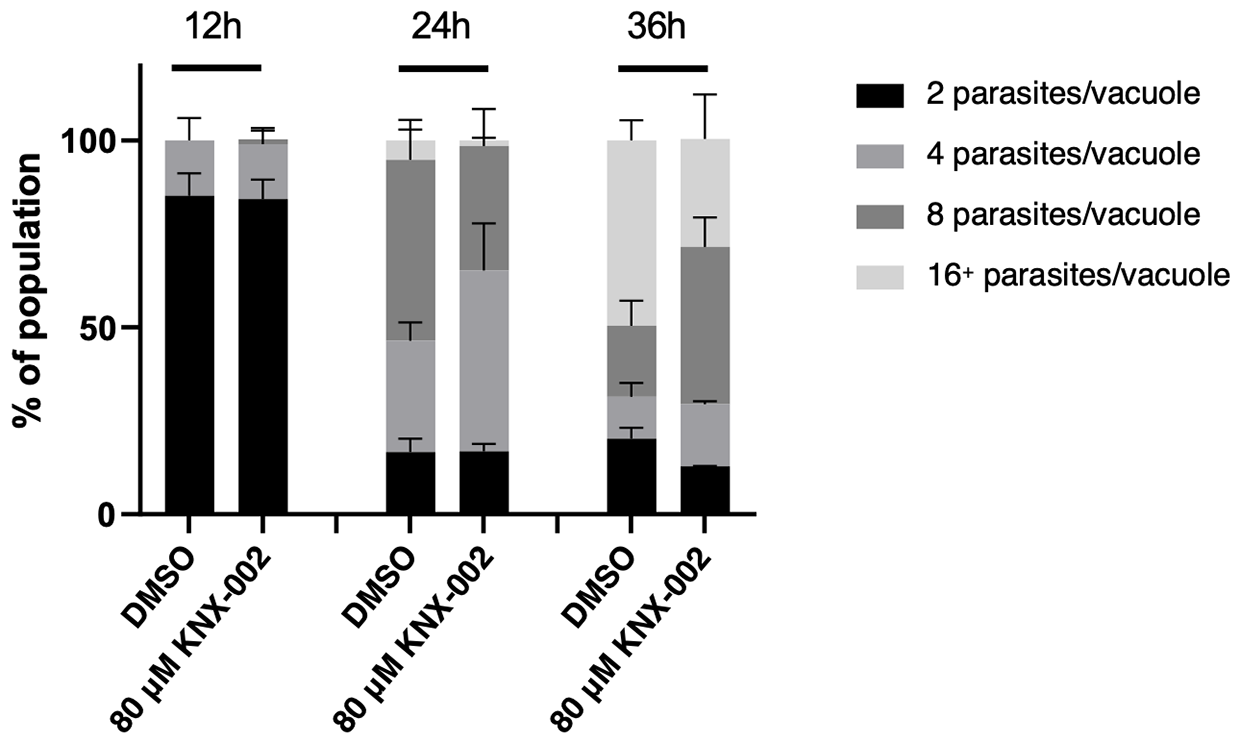

Supplement: S2 Fig — Newly infected HFF cells were incubated at 37°C in culture medium containing 80 μM KNX-002 or an equivalent volume of DMSO (vehicle) for the indicated times. The cells were then fixed and processed for immunofluorescence using an antibody against the inner membrane complex marker IMC1, enabling the number of parasites in each of 100–120 vacuoles to be counted. Shading of the bars indicates number of parasites per vacuole (see key); the means ± SEM at each time point of 2–5 biological replicates (each consisting of 2 technical replicates) are shown. There were no significant differences between the number of DMSO- and compound-treated parasites per vacuole at each of the time points, based on unpaired Student’s t tests using a 2-step Benjamini, Krieger, and Yekutieli correction for multiple comparisons. The measurements underlying the data plotted in this figure can be found in S3 Data. (TIF) [file pbio.3002110.s002.tif]

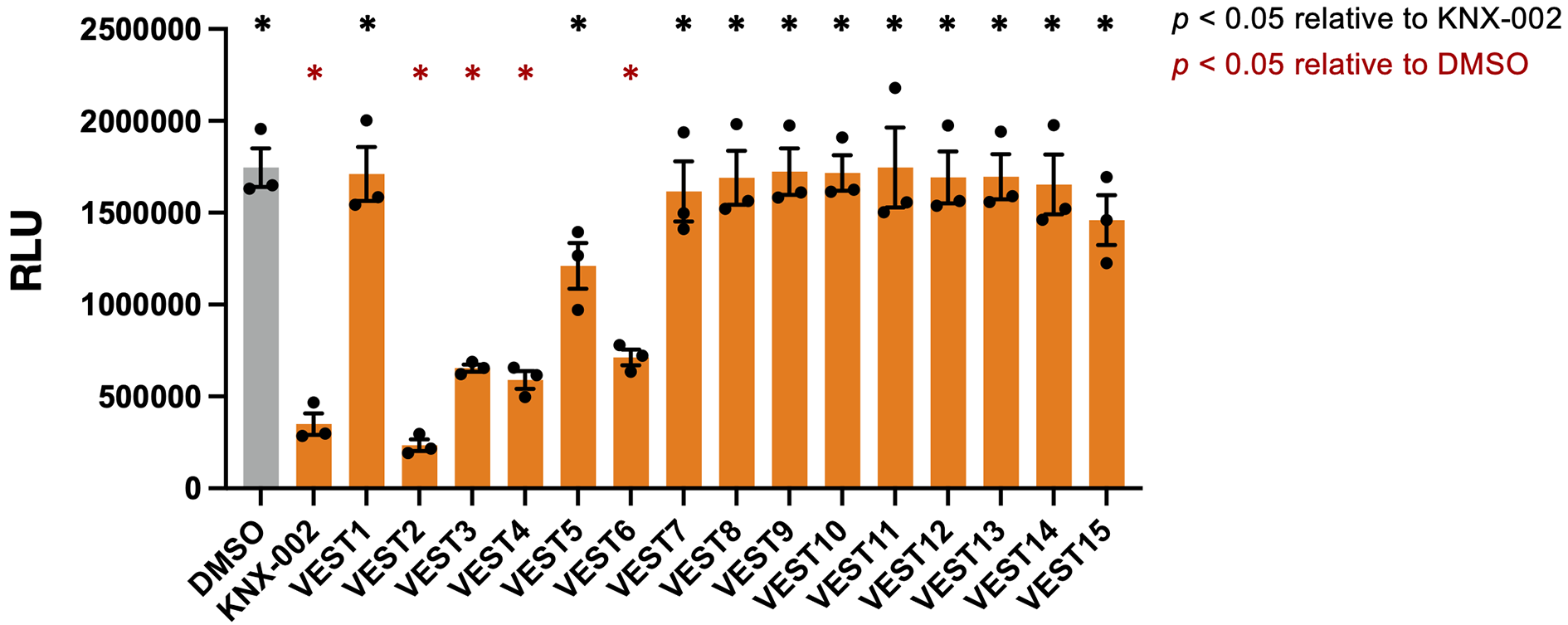

Supplement: S3 Fig — Actin-activated ATPase activity of wild-type TgMyoA in the presence of DMSO (vehicle), KNX-002, or each of the 15 KNX-002 analogs shown in Fig 3. All compounds were used at 20 μM. ATPase activity is expressed as relative luminescence units (RLUs). Each data point represents a single biological replicate composed of 2 technical replicates. Bars show the mean ± SEM of 2 biological replicates. RLU values were compared pairwise to KNX-002 (black asterisks) or to DMSO (red asterisks) by one-way ANOVA with Dunnett’s test for multiple comparisons; asterisks indicate p < 0.05. The measurements underlying the data plotted in this figure can be found in S4 Data. (TIF) [file pbio.3002110.s003.tif]

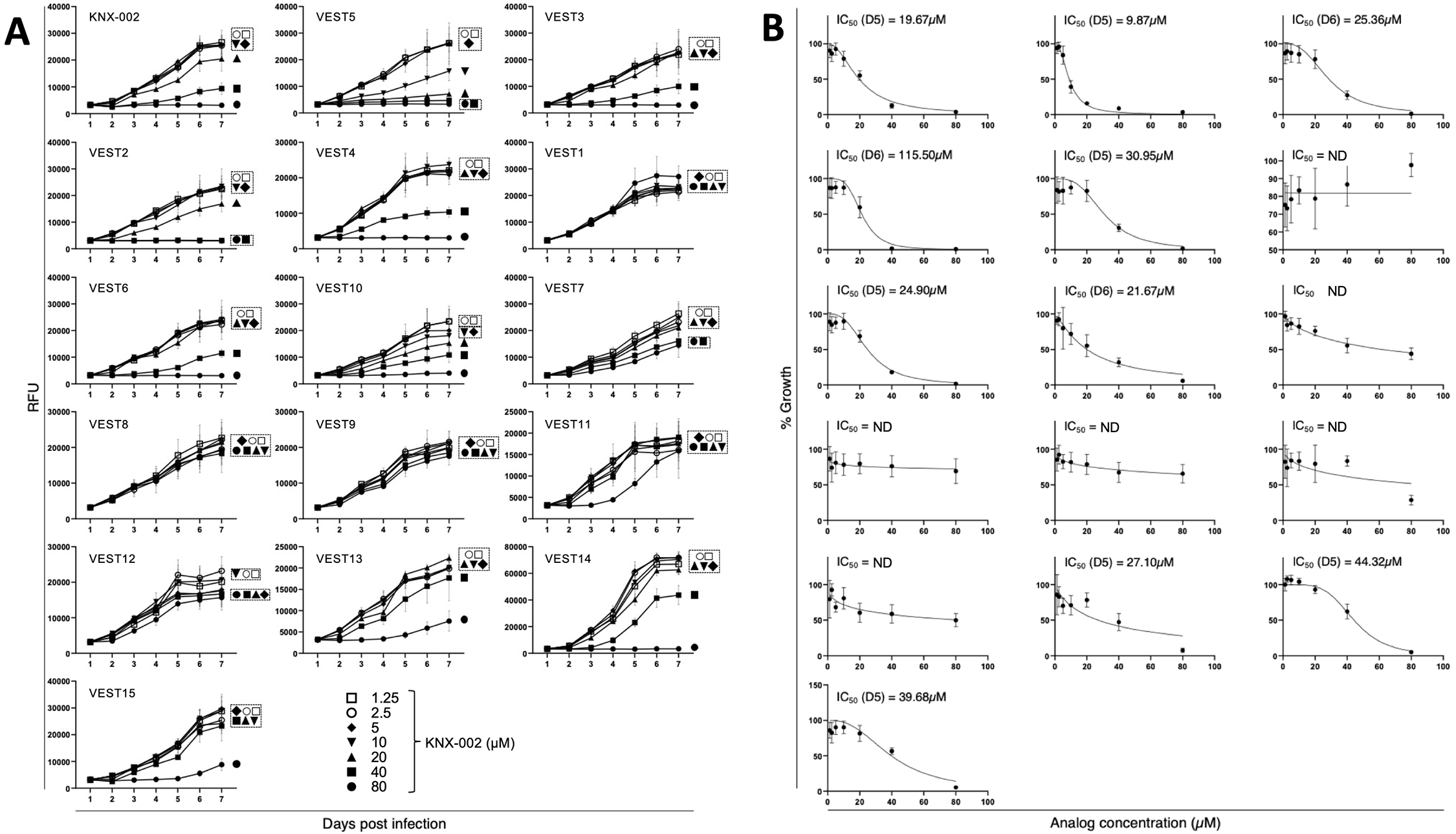

Supplement: S4 Fig — (A) tdTomato-expressing wild-type parasites were preincubated for 5 min with various concentrations (see key, bottom right) of KNX-002 or one of the 15 analogs shown in Fig 3 and then added to HFF cells on a 384-well plate. Fluorescence was measured daily over the next 7 days to quantify parasite growth. Each data point represents the mean of 3 biological replicates, each consisting of 2 to 3 technical replicates. RFU = relative fluorescence units. (B) The data from either day 5 (D5) or day 6 (D6) of the growth assay shown in panel A were used to calculate the IC50 for parasite growth; ND = insufficient inhibition from 0–80 μM to determine an IC50. The measurements underlying the data plotted in this figure can be found in S5 and S6 Data. (TIF) [file pbio.3002110.s004.tif]

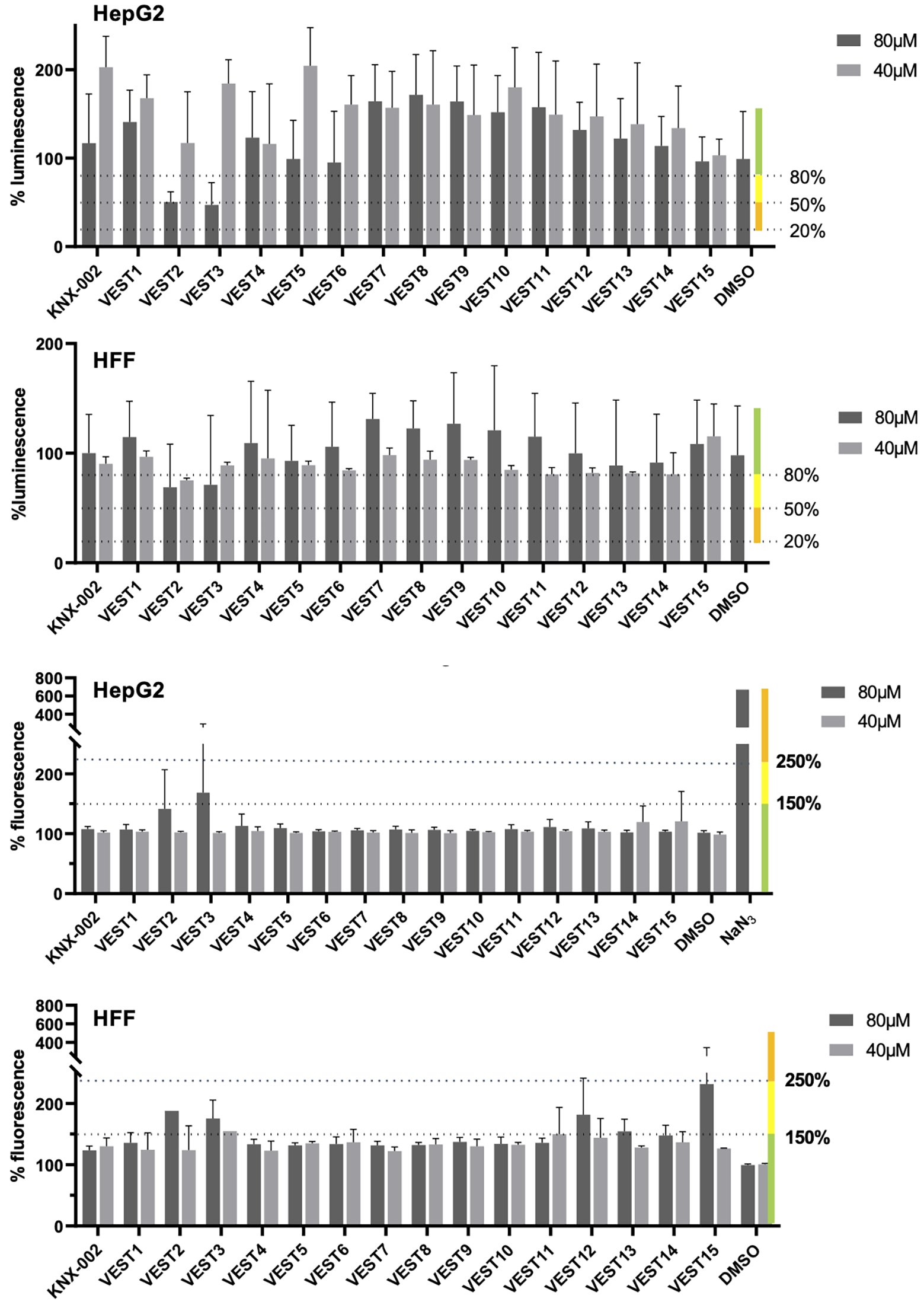

Supplement: S5 Fig — Subconfluent HepG2 and HFF cells were treated for 72 h with DMSO (vehicle), KNX-002, or each of the 15 KNX-002 analogs shown in Fig 3, at a final concentration of either 40 μM (light gray bars) or 80 μM (dark gray bars). The treated cells were then subjected to CellTiter-Glo viability (top 2 panels) and CellTox green cytotoxicity assays (bottom 2 panels). Colored bars on the right indicate thresholds for negligible (green), mild (yellow), and moderate (orange) loss of viability (top 2 panels) or toxicity (bottom 2 panels). The measurements underlying the data plotted in this figure can be found in S7 Data. (TIF) [file pbio.3002110.s005.tif]

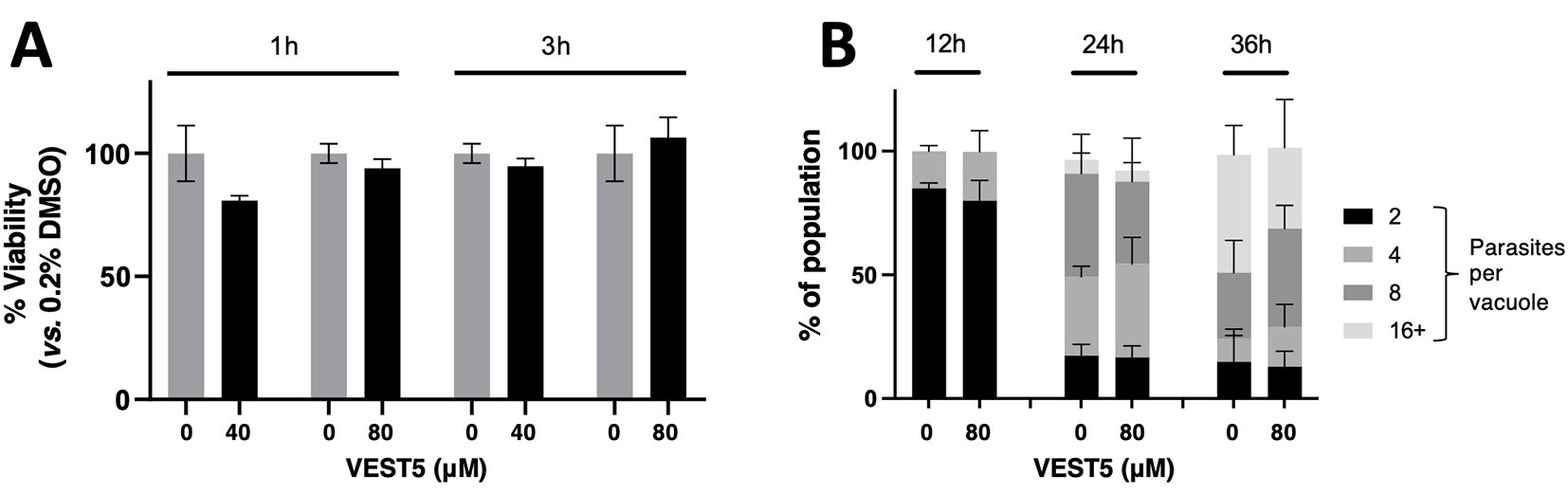

Supplement: S6 Fig — (A) Extracellular parasite toxicity. Wild-type parasites were incubated at 37°C in culture medium containing 0 (DMSO only), 40 μM, or 80 μM VEST5 for the indicated times. Parasite viability was then determined using the CellTiter-Glo assay. Bars show the mean of 2 biological replicates ± SEM. (B) Intracellular parasite toxicity. Newly infected HFF cells were incubated at 37°C in culture medium containing 0 (DMSO only) or 80 μM VEST5 for the indicated times. The cells were then fixed and processed for immunofluorescence using an antibody against the inner membrane complex marker IMC1, enabling the number of parasites in each of 50 vacuoles to be counted. Shading of the bars indicates number of parasites per vacuole (see key); the means ± SEM at each time point of 2 biological replicates are shown. The measurements underlying the data plotted in this figure can be found in S8 Data. (TIF) [file pbio.3002110.s006.tif]

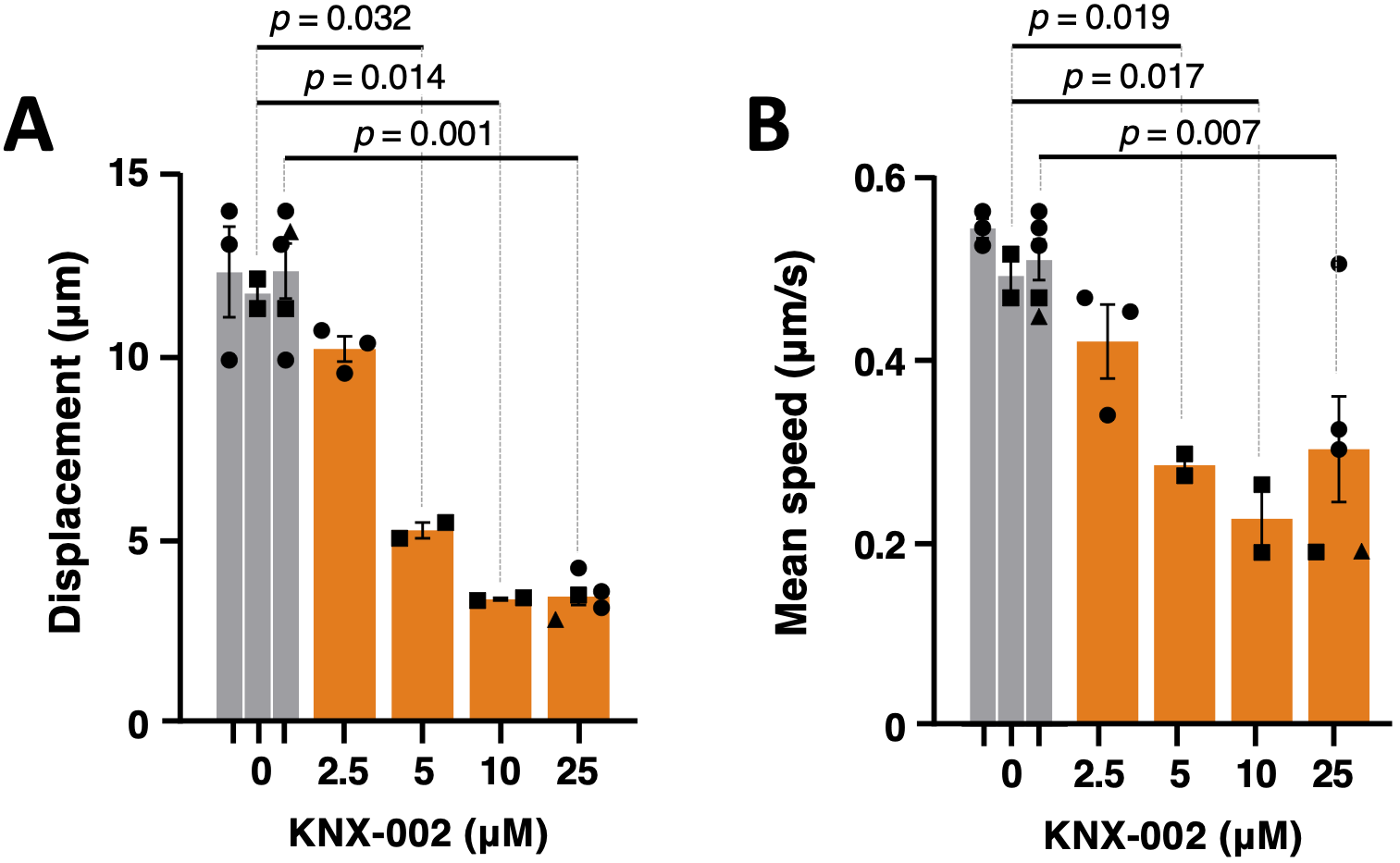

Supplement: S7 Fig — (A) First-to-last point trajectory displacement and (B) mean speed along the trajectory during a 60 s 3D motility assay in the presence of the indicated concentrations of KNX-002. Each data point represents a single biological replicate composed of 3 technical replicates. The number of trajectories included in the displacement and speed measurements decreases with increasing concentration of compound due to the reduction in percent moving (see Fig 4C). Sets of DMSO- and compound-treated parasite data captured on the same days, indicated by the similar symbol shapes, were compared by Student’s one-tailed paired t tests. Bars show the mean of the biological replicates ± SEM. Only the statistically significant differences (p < 0.05) are indicated. The measurements underlying the data plotted in this figure can be found in S9 and S10 Data. (TIF) [file pbio.3002110.s007.tif]

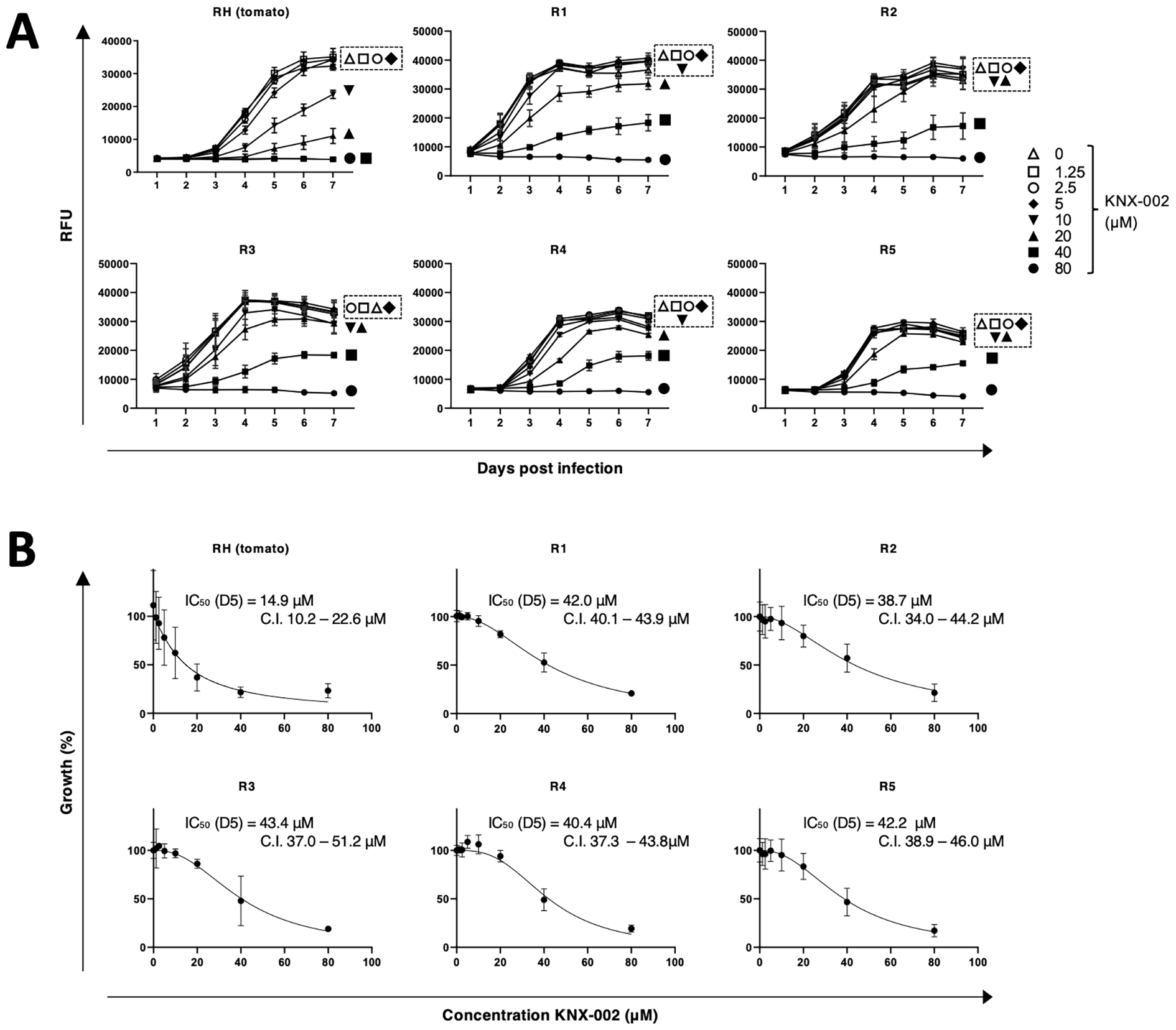

Supplement: S8 Fig — (A) tdTomato-expressing were mutagenized, selected in 40 μM KNX-002, and cloned as described in Methods. Each of the 26 clones recovered from 2 independent rounds of mutagenesis and selection was preincubated with various concentrations of KNX-002 for 5 min and then added to HFF cells on a 384-well plate. Fluorescence was measured daily over the next 7 days to quantify parasite growth. RFU = relative fluorescence units. (B) IC50 curves corresponding to the growth assay data shown in panel A, for the 5 clones that showed an IC50 shift of >2.5-fold compared to the un-mutagenized RH (tomato) parasites. The data shown are from 3 independent biological replicates, each consisting of 2 to 3 technical replicates at all time points. Vertical bars indicate SEM in panel A and 95% CI in panel B. Clone R3 contains a T130A mutation in the gene encoding TgMyoA; none of the other 4 clones contain mutations in the genes encoding TgMyoA, TgMLC1, TgELC1, or TgELC2. The measurements underlying the data plotted in in this figure can be found in S11 and S12 Data. (TIF) [file pbio.3002110.s008.tif]

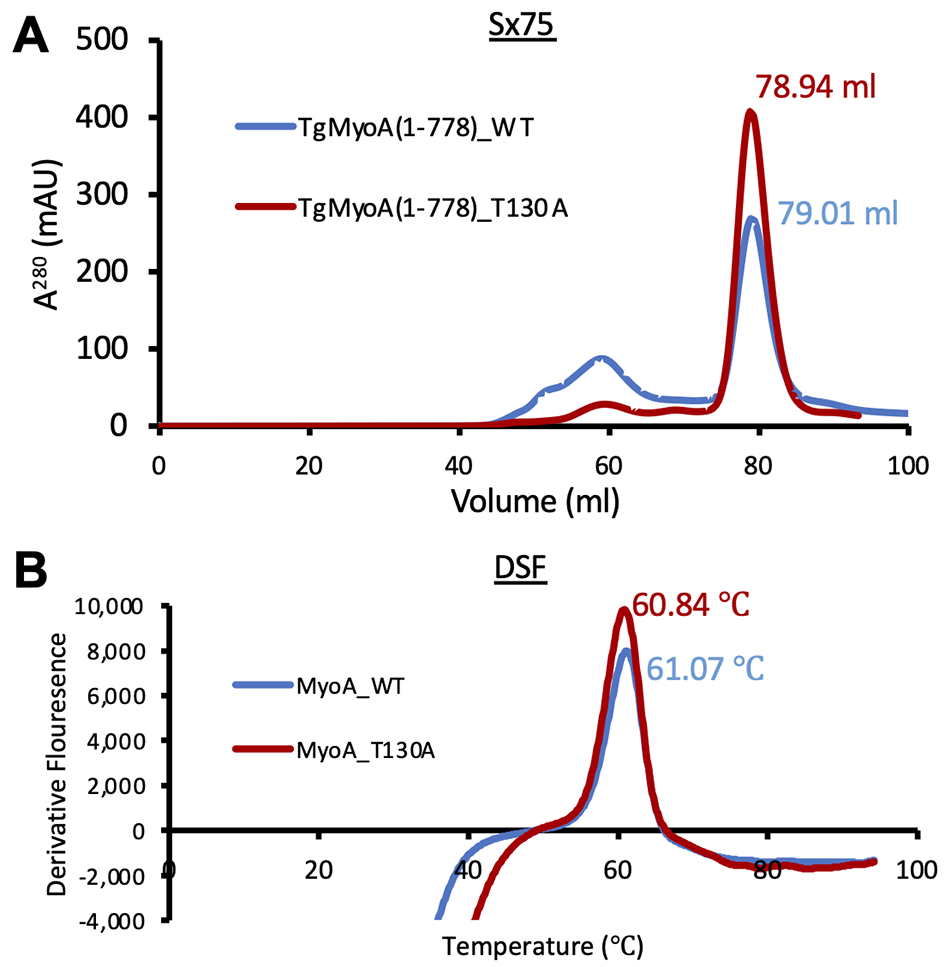

Supplement: S9 Fig — (A) SEC UV traces of TgMyoA motor domain, wild-type (blue), and T130A mutant (red), showing identical elution profiles. (B) Derivative fluorescence data from DSF experiment showing minimal changes in thermostability. Values are average of triplicate data. The measurements underlying the data plotted in this figure can be found in S14 and S15 Data. (TIF) [file pbio.3002110.s009.tif]

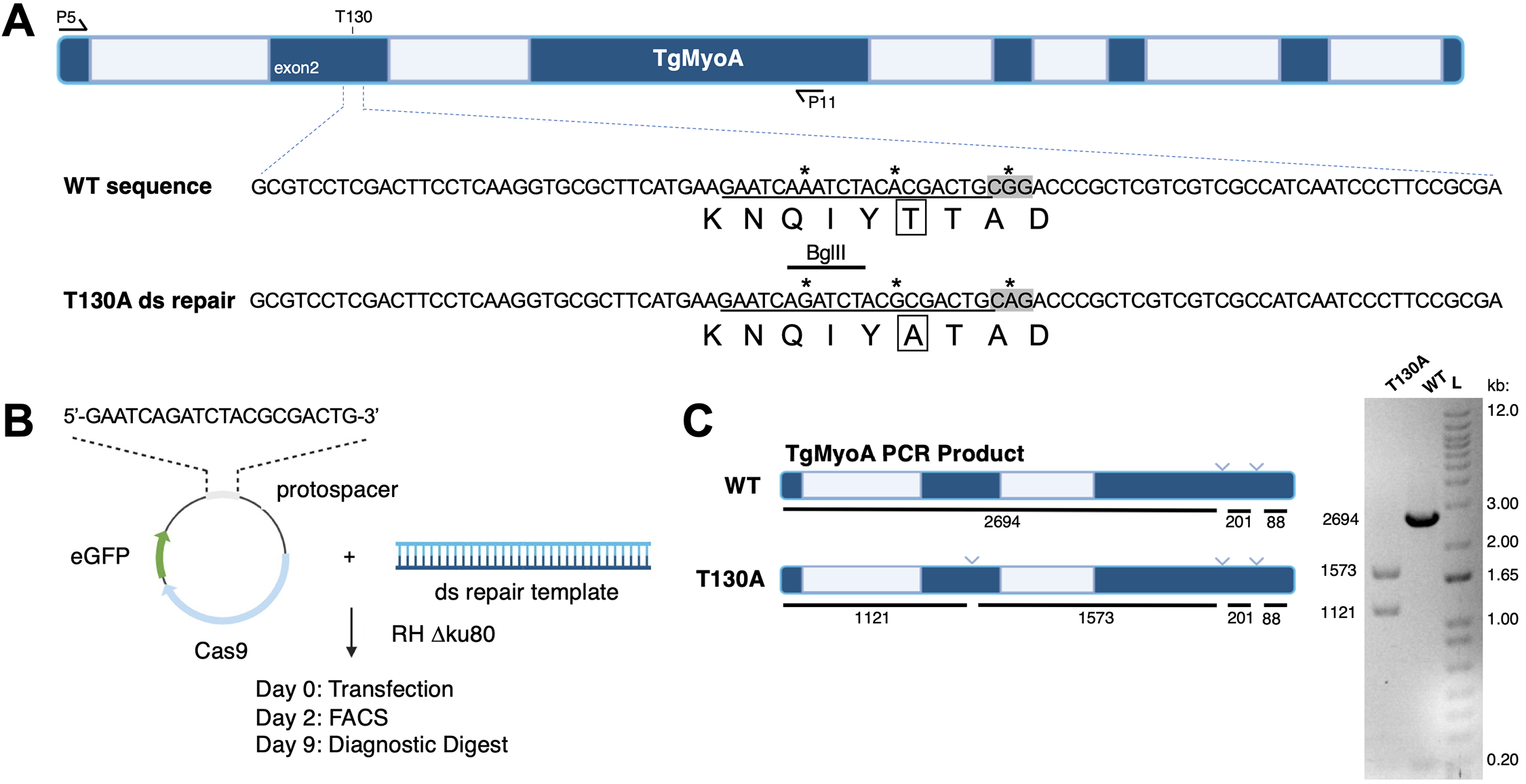

Supplement: S10 Fig — (A) Schematic of the TgMyoA gene (exons in dark blue, introns light blue) and the amino acids surrounding T130 (boxed) in exon 2. Mutations introduced by the double stranded (ds) repair template are denoted with asterisks (*): the leftmost mutation introduces a diagnostic BglII site, the middle mutation generates the T130A substitution (boxed), and the rightmost mutation ablates the PAM sequence. The guide sequence (protospacer) is underlined, and the PAM sequence is highlighted in gray. The location of primers P5 and P11 used to amplify a fragment from gDNA for diagnostic digestion (see panel C) are shown. (B) Timeline showing transfection of RHΔku80Δhxgprt parasites with the Cas9 GFP guide vector and ds repair template on day 0, sorting of eGFP+ parasites on day 2, and diagnostic digest of gDNA from individual eGFP+ clones on day 9. (C) Left schematic shows predicted fragment sizes after BglII digestion of the PCR fragment generated by P5/P11, for parental (WT) and T130A parasites. Right panel shows the BglII restriction digest of a T130A positive clone (leftmost lane) and WT clone (middle lane); the 201 and 88 bp fragments are not resolved on this gel. Right lane = ladder, selected band sizes indicated in kb. Clones that showed the expected digestion pattern for the T130A substitution were verified by sequencing using primers P5-P13 (S1 Table). The original uncropped DNA gel from which panel C is derived is shown in S1 Raw Images. (TIF) [file pbio.3002110.s010.tif]

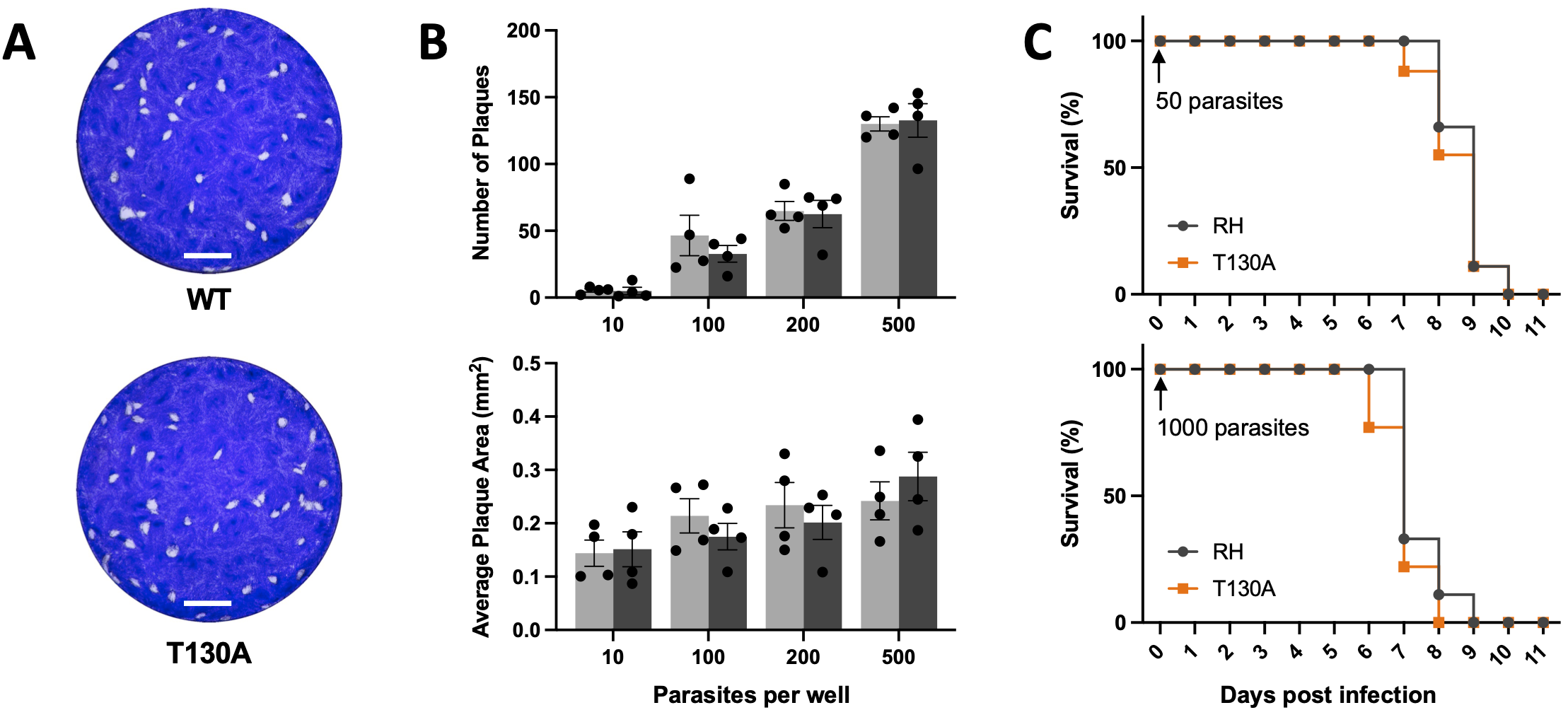

Supplement: S11 Fig — (A) Representative images of a plaque assay using wild-type (top image; WT) and T130A mutant (bottom image) parasites, 7 days after inoculating confluent HFF monolayers with 100 parasites/well. The entire well (22.6 mm diameter) from the 12-well plate is shown for each parasite line; scale bars = 4 mm. (B) Total number of plaques per well and average plaque area in mm2 of HFF monolayers inoculated with 10, 100, 200, and 500 parasites. The data represent the mean ± SEM from 4 independent biological replicates. There was no significant difference in plaque number or size between WT and T130A parasites at any of the inoculum sizes assayed. (C) Nine mice were infected on day 0 with 50 (top panel) or 1,000 (bottom panel) wild-type RH (black circles) or T130A mutant parasites (orange squares). There was no significant difference in survival over the next 11 days between mice infected with the same number of wild-type or T130A parasites (log-rank Mantel–Cox test). The measurements underlying the data plotted in panels B and C can be found in S16 and S17 Data, respectively. (TIF) [file pbio.3002110.s011.tif]

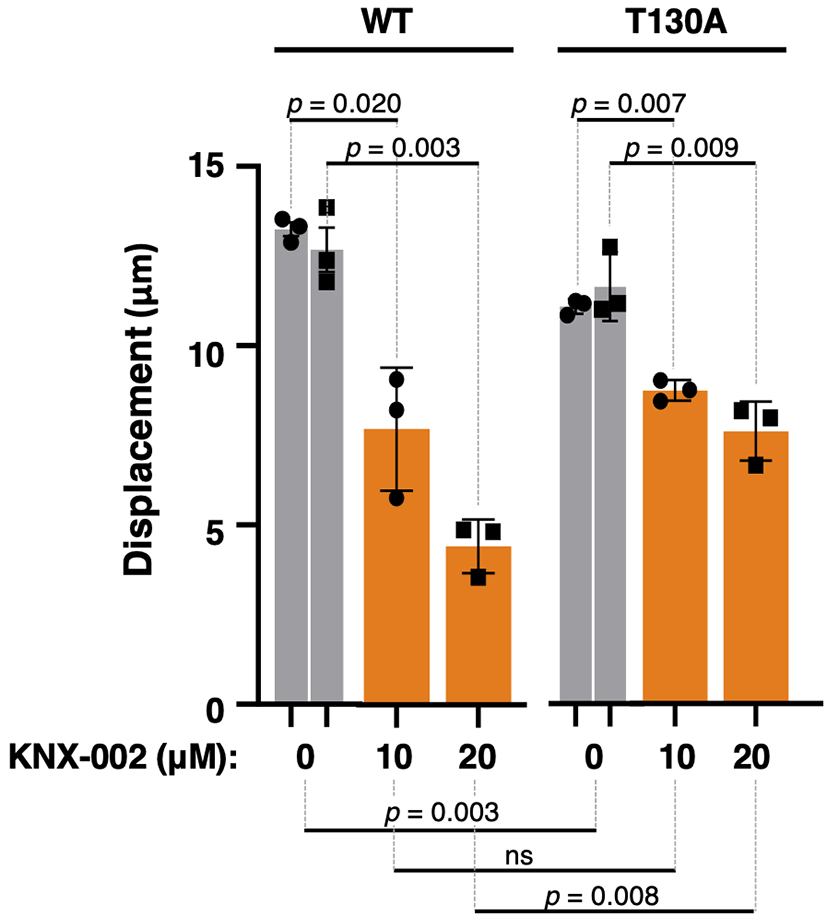

Supplement: S12 Fig — Displacement of wild-type (WT) and T130A mutant parasites during 80 s of motility in Matrigel, in the presence of 0 (DMSO vehicle only), 10, or 20 μM KNX-002. The data were derived from the same set of experiments shown in Fig 7B–7D. Each data point represents a single biological replicate composed of 3 technical replicates; 3 of the biological replicates were collected on the same 3 days (circles), and 3 of the biological replicates were collected on a different 3 days (squares). Bars show the mean of the biological replicates ± SEM. Sets of biological replicates using the same parasite line and collected on the same days were compared by Student’s one-tailed paired t tests (significance indicated above the graphs). Sets of biological replicates comparing different parasite lines were analyzed by Student’s two-tailed unpaired t tests (significance indicated below the graphs). ns = not significant. The measurements underlying the data plotted in this figure can be found in S18 and S19 Data. (TIF) [file pbio.3002110.s012.tif]

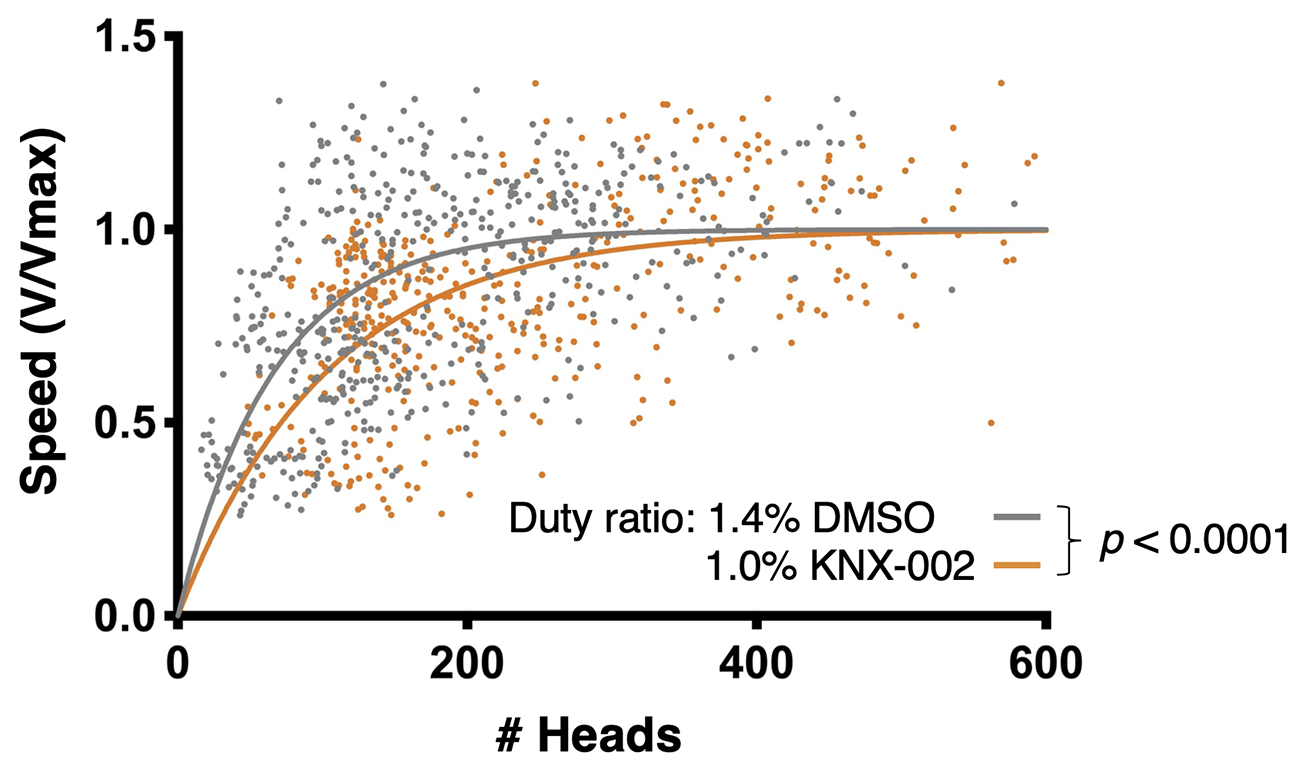

Supplement: S13 Fig — Actin filament velocity (V/Vmax) was plotted as a function of the number of myosin heads capable of interacting with the actin filament. The duty ratios calculated from these data (see Methods) were significantly different for TgMyoA treated with DMSO (gray datapoints) vs. 10 μM KNX-002 (orange; Kolmogorov–Smirnov test, p < 0.0001). The measurements underlying the calculated duty ratios shown in this figure can be found in S22 Data. (TIF) [file pbio.3002110.s013.tif]

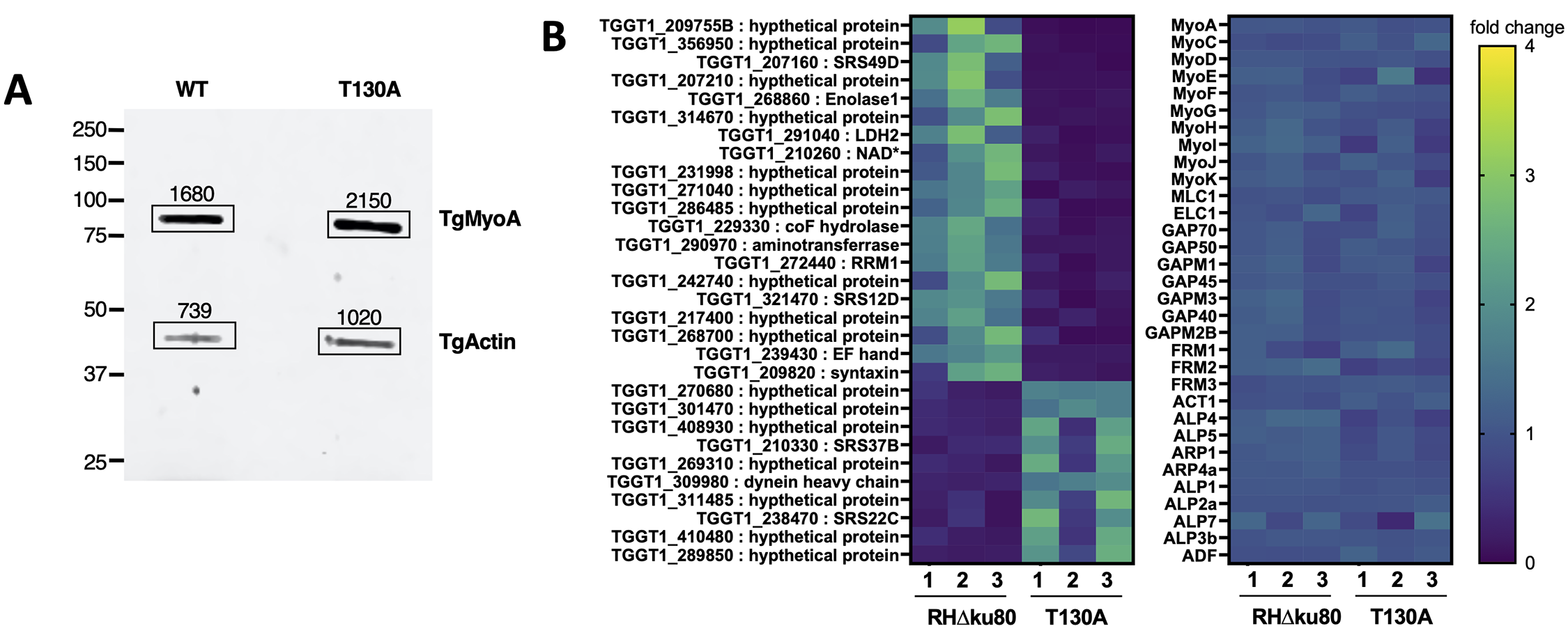

Supplement: S14 Fig — (A) The expression level of TgMyoA in wild-type (WT) and T130A mutant parasites was compared via quantitative western blotting. The blot was also probed for TgACT1 as a loading control; when normalized to the corresponding actin signals, the TgMyoA band intensities were within 7% of each other. (B) Left panel: The most differentially expressed genes between WT (RHΔku80) and T130A parasites (20 up-regulated and 10 down-regulated), after filtering for padj < 0.05 and FPKM > 0.1 and eliminating any genes that did not show the same pattern in all 3 replicates. Right panel: Analysis of differential expression of selected genes with the potential to compensate for loss of TgMyoA function, including other myosin motors, known MyoA-interacting proteins, glideosome components, actin and actin regulatory factors. The original uncropped western blot from which panel A is derived is shown in S1 Raw Images and the original expression data from which the highlighted results in panel B are derived are found in S23 Data. (TIF) [file pbio.3002110.s014.tif]

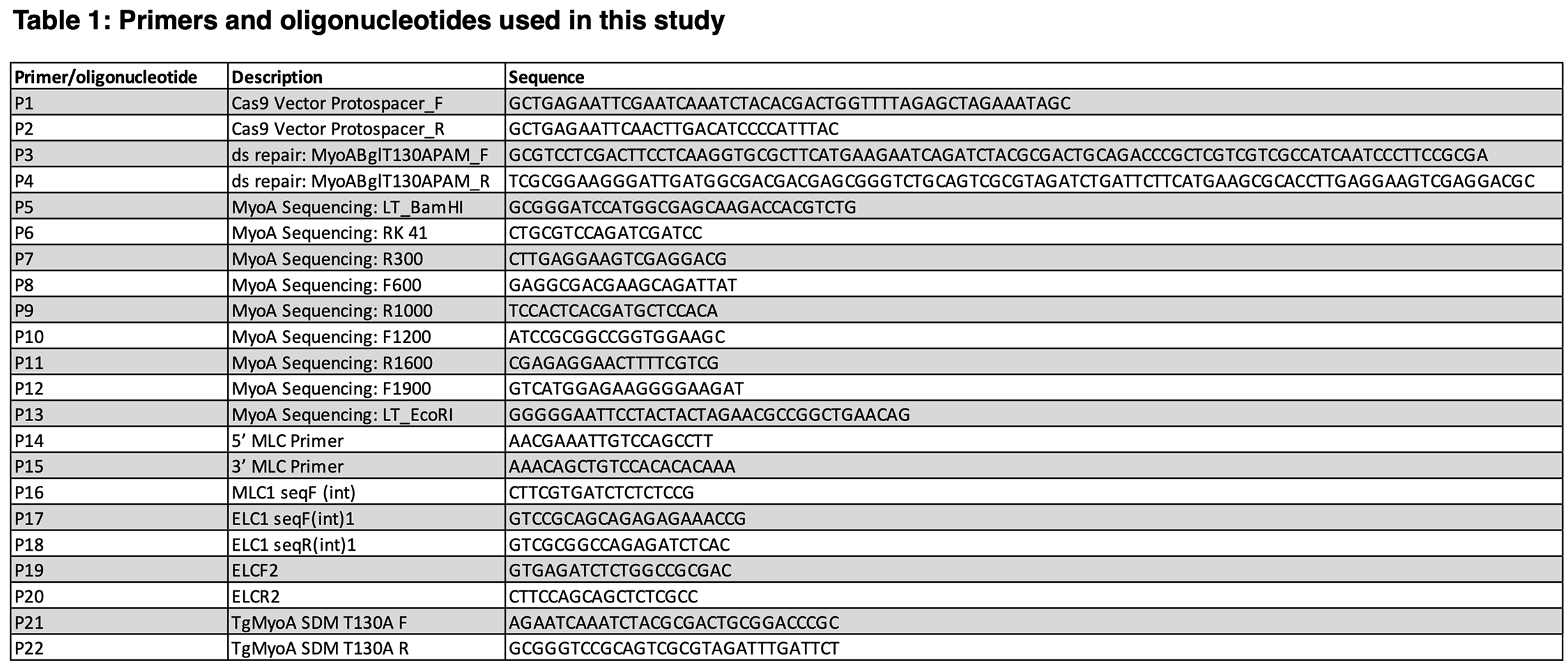

Supplement: S1 Table — (TIF) [file pbio.3002110.s015.tif]

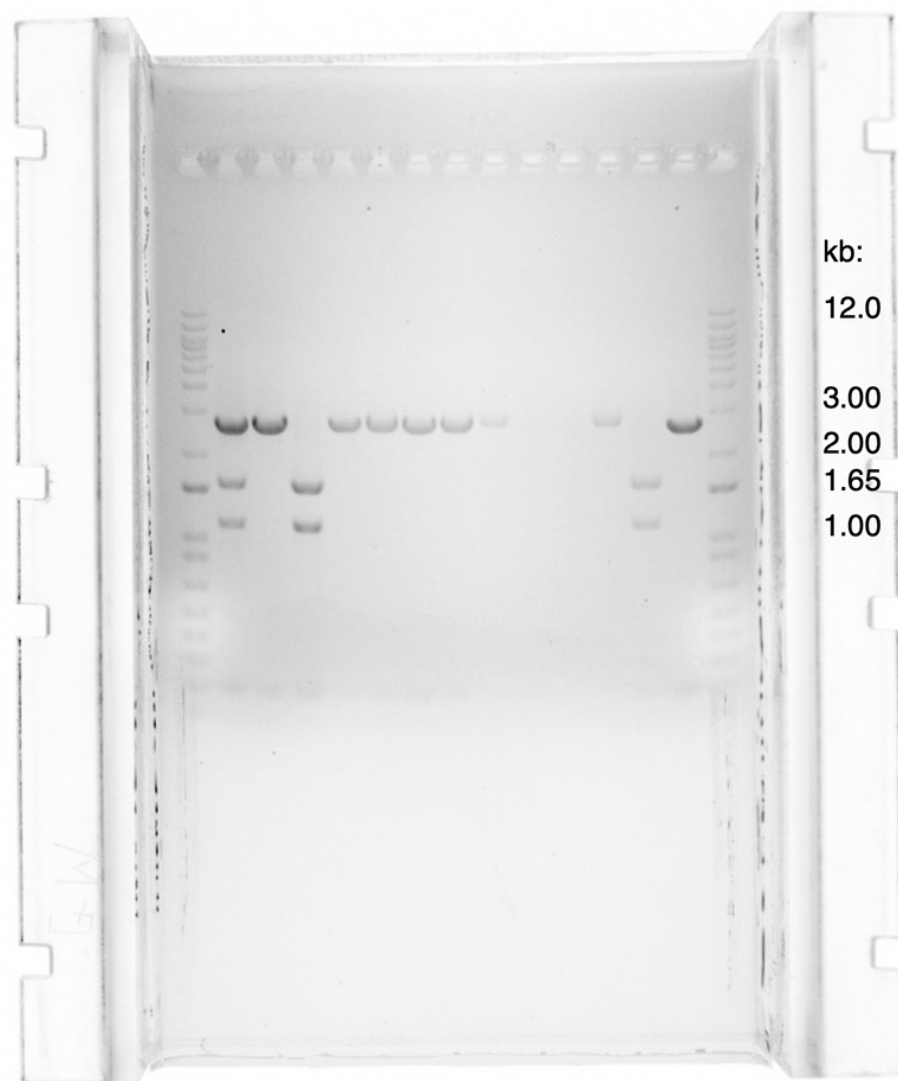

Unprocessed image of DNA gel shown in S10 Fig

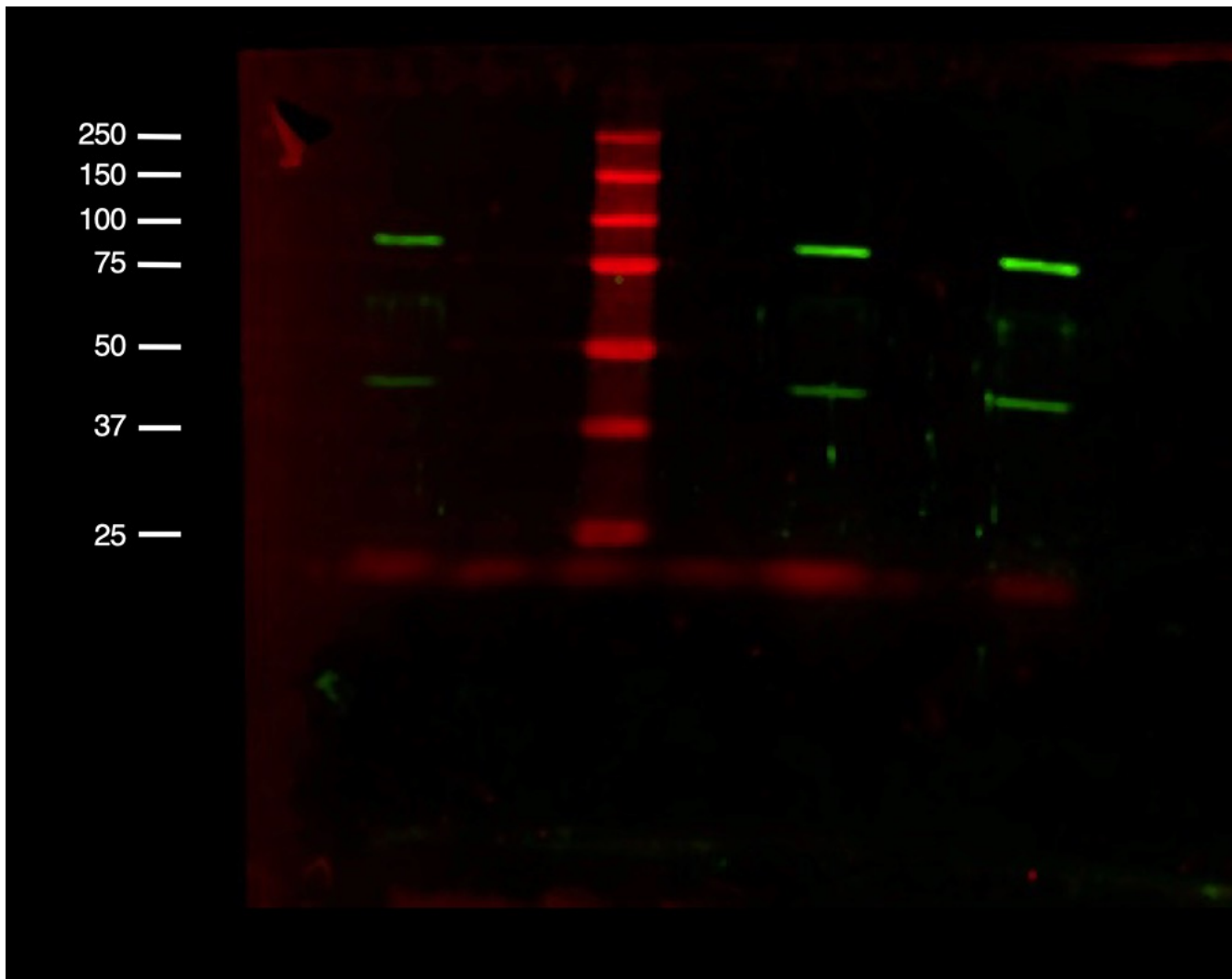

Unprocessed image of Western blot shown in S14 Fig

Supplement: S1 Raw Images — (PDF) [file pbio.3002110.s016.pdf]
