# Supplementary material for: MyosinA is a druggable target in the widespread protozoan parasite Toxoplasma gondii
Source: PLoS Biol. 2023 May 8;21(5):e3002110. doi: 10.1371/journal.pbio.3002110 (PMC10185354; doi:10.1371/journal.pbio.3002110)
Supplement: Attachment — Submitted filename: 131478_1_rebuttal_2594814_rq1vs8.pdf [file pbio.3002110.s041.pdf]

Full Revision

Review COMMONS

Manuscript number: RC-2022-01738
Corresponding author(s): Gary E. Ward

1. General Statements

We thank the reviewers for their thoughtful comments and suggestions, which have improved the manuscript. We are particularly gratified by their positive comments about the significance of the findings. Our point-by-point responses to the reviewer comments and suggestions are summarized below. Line numbers have been added to the revised manuscript to make it easier to locate the changes.

2. Point-by-point description of the revisions

Reviewer #1

1) In the study there is a lack of consideration of other targets. In and of itself this is not a problem, but once the author's identified the T130A mutation as being a key for protection it would have been good to Sanger sequence the other T. gondii myosins - a quick alignment of the TgMyo's A, C, H (class XIV), along with D and E suggests that the motif is highly conserved. This raises the currently unexplored (and exciting!) prospect of a pan-myosin inhibitor, and that there might have been mutations at an equivalent position in the other four KNX002 resistant clones - for example, MyoC has been proposed to provide some level of functional redundancy in the absence of MyoA.

> Because the goal of this work was to evaluate the druggability of TgMyoA, we specifically designed our experiments to identify resistance-conferring mutations in TgMyoA or its light chains, as described on lines 366-372. This strategy yielded the TgMyoA T130A mutation, which enabled us to rigorously determine that inhibiting TgMyoA, and TgMyoA only, was sufficient to slow the progression of disease *in vivo*. Because we took this targeted approach, our results did not address: (a) the basis of resistance in the 4 resistant clones that did not contain a mutation in TgMyoA or its light chains and (b) whether KNX-002 inhibits any of the other ten parasite myosins.

The most informative way to address (a) would be to do whole genome sequencing on each of the mutants, since resistance might have nothing to do with the other parasite myosins or their light chains. Any potential resistance-conferring mutations identified would need to be regenerated in a non-mutagenized background and functionally characterized, as we have done here for the T130A mutation, to be certain that this particular mutation was responsible for resistance. The most direct way to address (b) would be to individually express each of the other ten parasite myosins together with its specific associated light chains and the myosin co-chaperone protein TgUNC, purify the motor and determine the effect of the compound on motor activity (as we have done for TgMyoA; Fig. 1). These are both major undertakings that are

beyond the goals and scope of the current manuscript. Critically, the absence of such data does not impact the conclusions of our phenotypic studies, which used the CRISPR-engineered T130A parasite line.

We nevertheless agree with the reviewer that these are both interesting questions that should be studied further, and we now discuss them on lines 416-422 and 451-453.

2) The fact that T130 is not thought to be the binding site of KNX002 is only introduced quite late on - this also relates to the next point - is the binding pocket conserved??? It is intriguing that the residue and proximal amino acid environment are highly conserved with vertebrates, but that KNX002 does not have an effect on their activity in their screen assay. It would be useful to know if the differences in the structures of the myosins can provide an explanation for this - along the same lines, given that the crystal structure of TgMyoA is available (PMID: 30348763), it would be useful for the authors to provide a molecular model for the binding of the inhibitor to the proposed point of engagement.

> These are excellent questions that we unfortunately cannot yet answer. Docking simulations of KNX-002 to the published structure of TgMyoA in its pre-powerstroke state have thus far not yielded any promising results. The site of KNX-002 binding to *P. falciparum* MyoA was determined by X-ray crystallography (ref. 54); however, the PfMyoA in that study was in the post-rigor state and the coordinates of the co-crystal structure have not yet been made available in the PDB database for homology modeling.

The lack of effect of KNX-002 on the vertebrate muscle myosins may not be surprising. Although the 3D structures of myosins are rather conserved, their primary structures are quite different, which likely contributes to the different effects of KNX-002 on the different myosins. TgMyoA and PfMyoA are more similar to each other than they are to the vertebrate muscle myosins, which may enable the specific targeting of MyoA in apicomplexan parasites (lines 308-310).

If this is an allosteric site it is possible that the mutation functions indirectly upon binding of KNX002 to the orthosteric site, but this would be useful to help the reader to understand this (and there are bioinformatic prediction tools that will score allostery - which would be interesting to include). This is explained somewhat in the discussion - but this should be introduced much earlier to clarify. What is known about allosteric regulation of Myo function? Is this a known site of regulation?

> Allosteric modulation of human cardiac myosin by small molecules such as omecamtiv is well established, and allosteric effects of the T130A mutation are certainly a possibility. As molecular motors, myosins depend on a complex and highly interconnected network of allosteric interactions to perform their function (for example, see ref. 59). This complexity, combined with the fact that the data on the PfMyoA-KNX-002 structure have not yet been released, makes it very difficult to generate any sort of model that would support meaningful conclusions. A

statement to this effect has been added to the Discussion (line 375-382), and the likelihood that T130 is not part of the actual binding site for the compound is now mentioned at the very beginning of the paragraph that discusses the potential mechanism of action of the T130A mutation (lines 375-376).

3) Introduction: 'Nearly one third of the world's population is infected with the apicomplexan parasite' - given that these data are extrapolated from serology, this should be reworded - it's fairer to say that they 'are or have been infected...'

> Done – line 65.

4) Page 4: figure 1A - can you provide some explanation for incomplete inhibition in the screen - there seems to be a residual amount (15-20%) of activity that is not inhibited.

> The compound inhibits 80-90% of the motor's activity at 40uM. We did not test higher concentrations in the ATPase assay; presumably we would see incrementally more inhibition as we increase compound concentration further, but the concentrations used enabled us to construct a reproducible IC_{50} curve without adding potentially confounding amounts of DMSO (carrier) to the assay.

5) The authors demonstrate a general effect on growth over 7 days. It would be good to use a replication assay (e.g. parasites/vacuole over a single lytic cycle) to confirm that KNX002 does not affect cellular division. This would further strengthen the argument that the phenotypic effect is primarily via impacts on motility.

> A figure showing the lack of effect of KNX-002 on replication has now been added (new Supplemental Figure 2) and a paragraph describing these data and their implications added to the Results section (lines 137-143).

6) Page 5: 'selected for parasites resistant to KNX-002 by growth in 40 µM KNX-002.' - could the authors add text to explain why that concentration was chosen.

> 40 µM is close to the compound's IC_{90} of 37.6 µM and, although we tried a number of different compound concentrations and selection schemes, 40 µM yielded parasites with the greatest shift in IC_{50}. We now include this rationale on lines 606-607, as well as a new figure showing the shift in IC_{50} curves for all 5 resistant lines (Suppl. Figure 8).

7) Page 6: 'suggesting that the effects of the T130A mutation on motor function are due to more subtle structural changes' - it's fair to say that there are not gross structural changes based on the data presented, but that does not mean it is therefore a 'more subtle structural change' - surely the mutation could prevent KNX002 binding without effecting TgMyoA structure?

> Based on the residues within *P. falciparum* MyoA that participate in binding to KNX-002 (ref. 54), it is unlikely that T130 of TgMyoA participates directly in compound binding. Mutation of T130 to alanine therefore seems most likely to impact compound binding through a change in protein structure, discussed more fully now on lines 375-382.

8) Page 6: 'the proportion of filaments moving' - in the figure it's referred to as the 'fraction of filaments', which makes more sense for the data presented. Please correct to 'fraction' throughout the manuscript (discussion, page 9 - possibly other instances!). Along the same lines, in figure 6 it would be good to change the y axes on the '% moving' to be 'fraction moving' and change the numbers - this would make it easier for the reader to understand the index values presented in the lower panels - if you do the calculation with the % values presented the numbers don't make sense (as fractions they do). The axes for motility also go up to 125% - please correct - based on the data presented there is no need for this to be above 100% (or 1 - see above).

> We thank the reviewer for this suggestion; "percent moving" and "proportion moving" have been changed throughout the manuscript and figures to "fraction moving". The y-axis labels on the motility and IC_{50} curves have also been modified as suggested.

9) Page 7: 'tested whether KNX-002 (20mg/kg, administered intraperitoneally on the day of infection and two days later' - please provide some rationale for the concentration used.

> A preliminary dose tolerance study was conducted prior to the infection experiments, with doses ranging from 5-20 mg/kg. The study showed that two doses of 20 mg/kg, administered two days apart, resulted in minor hepatoxicity without signs of pain or distress. 20mg/kg was therefore considered the maximal tolerated dose. This rationale is now included on lines 715-721.

10) Page 10: 'the T130A mutation is likely to have long range structural impact that could alter the KNX-002 binding pocket' - this is particularly interesting, and should be addressed with a model - do the authors think that the T130 region be a conserved site of allosteric regulation? This would be good to expand upon in the discussion - mutation of an allosteric site as a mechanism of resistance is unusual, and typically described as being unlikely - and used as justification for the targeted drugging of allosteric sites.

> See response to comment #2 above and the new text on lines 375-382.

Reviewer #2

1) Considering (i) the moderate effect of KNX-002 on the acute infection process in CBA mice that received tachyzoites intraperitoneally, (ii) the fact that the drug application cannot be envisaged outside of the context of reactivation of cystogenic strains (in particular with respect to cerebral toxoplasmosis as emphasized in the introductive section), which implies the drug would have to be delivered and active in the brain parenchyma, a condition not analyzed here, it would be appropriate to modify the current title. It would be more relevant to highlight the solid body of data on the identification and functional characterization of the compound and derivatives in vitro and in the host mouse model. Apart from the title, the discussion should also recontextualize the in vivo assays and the information these assays bring on the slight delay of the "mortality" of some but not all mice.

> We agree that the major clinical application of any new anti-Toxoplasma chemotherapy would be treatment of a reactivated infection, particularly in the brain (although there could also be a role for treatment of pregnant women), and that the data we present with this compound do not speak directly to clinical efficacy in this context. That said, reactivation leads to an active infection whose pathogenesis requires TgMyoA-dependent motility, invasion and egress, like the active infections analyzed here. The KNX-002 scaffold would likely need to be modified to enable it to cross the blood-brain barrier and access parasites in the brain, but that would be a normal step in any campaign to develop new drugs for toxoplasmosis (which is well beyond the scope of this study; see response to comment #11).

Given these considerations, we gave much thought to how to accurately describe the results from the animal experiments – and we therefore appreciate the reviewer's comment. For the title, we arrived the word "druggable", because it has the very specific meaning described on lines 100-101: a protein whose activity is amenable to inhibition by small molecules. In our experiments with mice infected with wild-type parasites, nine of the ten compound-treated animals survived longer than the untreated controls, and 40% of the treated mice were still alive at the end of the experiment. Nevertheless, we stayed away from terms like "therapeutic" or "treatment", for exactly the reasons the reviewer raises. We believe that the current title is an accurate summary of what we found, since we have indeed shown that MyoA is amenable to inhibition by a small molecule in a well-established animal model of infection (CBA mice infected intraperitoneally). Showing for the first time that the MyoA is druggable, *in vivo*, provides the rationale for identifying more potent compounds that can access the brain and serve as *bona fide* leads for drug development.

To the reviewer's point, we also reviewed all sections of the text where we described the animal experiments, and in the revised manuscript we replaced all instances in the text of "ameliorate disease", "prevent disease" and "decrease the susceptibility of mice to a lethal infection" with the more circumspect phrases "alter disease progression" or "slow disease progression" (lines 46, 56, 110, 297, 315, Figure 9 legend). We also changed the title of the Results section describing these data from "KNX-002 treatment decreases the susceptibility of mice to lethal infection with *T. gondii*" to "KNX-002 treatment slows disease progression in mice infected with a lethal dose of *T. gondii*" (line 284).

2) Motility analysis: This comment concerns the Figure 7. It seems to the reviewers that the major hypothesis to test in data presented in panel B is that the wild type and the T130A mutant tachyzoite respond differently under similar drug conditions rather than the two populations without drug. These statistics could be added easily, hence it would validate that the proportion of motile mutant parasites is not affected by the drug when compared to vehicle.

> These statistical comparisons have now been added to revised figure 7, as suggested. Since this comparison was between different parasite lines, it required the use of unpaired t-tests (*vs*. the paired t-tests used for different compound treatments of the same parasite line). We have therefore revised all 3D motility figures (Figures 4 and 7, Suppl. Figures 7 and 12) and their legends to clearly indicate which samples are being compared to which and whether paired or unpaired statistical tests are being used.

However, the statistics shown panel C rather suggest that the drug does impact on the speed of the moving parasites, including when these carry the "resistance" T130 A mutation. It is not clear what we can gain in terms of messages with the motility index except to "slightly reverse" the analysis on panel B and to favor a no-effect of KNX-002 on the mutant parasite motile skills, on which the author might give more explanation. When comparing these quantitative tests with the panel presented above (panel A) it seems that the mutant parasite is still impacted by the MyoA inhibitor. Although there is no doubt for the reviewers that the T130A mutant emerging from the selected T. gondii resistant clones is a valuable probe for assessing drug selectivity: indeed the assays validate KNX-002 as a direct TgMyoA ATPase inhibitor, it might be good to rephrase some sentences and to have a harmonized definition of the parasite motility index throughout the text (Figure 7 legend, result and discussion sections).

> The reviewer is correct that there is a decrease in the speed of compound-treated T130A parasites, as the *p*-values on Figure 7C indicate. This is why we state in the text that "the mutant parasites retain some sensitivity to the compound" (line 263). We were careful throughout the manuscript to refer to the resistance provided by the mutation as "partial", or to describe it as a "reduced sensitivity". Partial resistance is still sufficient to establish compound specificity, as noted by the reviewer in this comment.

We present the motility index not to try to "reverse" the effect of the compound on the mutant's speed, but because the compound has two simultaneous effects on motility -- a decrease in the fraction moving and a decrease in speed of those that do move. Combining these two effects into one value (while still showing each component individually, as we have) enables comparison to the analogous actin filament motility index from the *in vitro* motility assays, and provides a more complete picture of the impact of compound treatment on parasite motility. This is a similar approach to that used in studies of *e.g.*, phagocytosis, where the widely reported "phagocytic index" corresponds to the fraction of cells that have internalized at least one particle multiplied by the average number of beads internalized. The motility index of the

mutant parasites is significantly less impacted by KNX-002 than the motility index of wild-type parasites (Figure 7D).

We have further clarified the definition and rationale for using the parasite motility index throughout, as suggested (lines 233-235, 264-267, 345-348).

This reviewer's concern was accentuated by the comparison between the actin filament sliding index and the parasite motility index which appears as such far stretch; Aside from the "far stretched claims" easy to re-address in a revised version, the readers have appreciated the writing quality and most figure illustration. The discussion nicely synthetizes the whole dataset, including those related to the 4 T. gondii clones that resisted to KNX-002 but not through mutations targeting any of the myosinA chains.

> We have added additional text to the discussion listing possible reasons for the differential effects of the mutation on the filament and parasite motility indices (lines 403-406).

4) Abstract: the concept of "ameliorate disease" in this framework is odd and the objective of the work can be rephrased in a simple way (see below)

> See response to Comment #1; "ameliorate" has been replaced with "alter disease progression" (line 46).

5) Introduction section: we think that the references on the impairment of invasiveness for the KoMyoA should be included (Bichet et al., BMC Biology 2016) as it has provided proof of an alternative and suboptimal mode of entry in many different cell types, thereby arguing that in absence of MyoA function, parasite invasiveness is not fully abolished and this without considering any MyoC-driven MyoA compensation.

> We thank the reviewer for catching this oversight; the Bichet citation has been added (line 93).

6) Introduction, third paragraph: in the sentence "Because the parasite can compensate for the loss or reduced expression of proteins important to its life cycle [29-31], small-molecule inhibitors of TgMyoA would serve as valuable complementary tools for determining how different aspects of motor function contribute to parasite motility and the role played by TgMyoA in parasite dissemination and virulence ». We definitively agree with this view but saying that, we think it would be worth evaluating (or simply discussing) the potency of the KNX-002 against MyoC, which compensatory contribution has been debated and remains questionable (at least to the reviewers) with respect to cell invasiveness restoration (related to the comment above).

> We have included a discussion of a potential compensatory role for MyoC and the value of determining in future studies whether KNX-002 (or its more potent downstream analogs) inhibit

any of the other parasite myosins (lines 419-423). Whether or not MyoC can functionally compensate for a lack of MyoA – we agree this is a controversial question – it is important to note (as we do on line 440-442) that "*T. gondii* engineered to express low levels of TgMyoA … are completely avirulent [28], arguing that sufficiently strong inhibition of TgMyoA is likely, on its own, to be therapeutically useful".

7) If we are correct, the screen and the characterization study have been performed with two different products (CK2140597 and KNX-002 the compound library and the re-synthetized one, respectively). Could we make sure that the two have the same potency?

> The source of compound used in each of the assays is now explicitly described on lines 481-490). Commercially obtained compound yielded an IC_{50} in growth assays of 16.2 and 14.9 µM (Figures 2 and 5, respectively), and compound synthesized by us yielded an IC_{50} value of 19.7 µM (Figure 3). The 95% confidence intervals of these three independent IC_{50} determinations with two different sources of compound overlap (lines 484-486).

8) We understood how the authors came to the conclusion that the KNX-002 impact on growth of the parasite and they stated "growth in culture" in the subsection title but then refers to parasite growth. Therefore, it looks a bit confusing for the reader since intracellular growth per se is probably not modified but this feature was not looked at it in this study (we would expect no impact based on published data on MyoA- genetically deficient tachyzoites, except if the drug impacts host cell metabolism for instances). Instead, it is the overall expansion of the parasite population that is analyzed here and clearly shown to be impacted. This decrease in population expansion on a cell monolayer likely results from impaired MyoA-dependent egress and invasiveness upon chemical inactivation of MyoA. Accordingly, it appears difficult to understand what is an IC50 for the "overall" growth in the context of this study. The authors should rephrase for better accuracy when necessary, including in the graph Fig2 legend axis.

> While assays that measure parasite expansion in culture are by convention called "growth" assays (e.g., see Gubbels et al, High-Throughput Growth Assay for *Toxoplasma gondii* Using Yellow Fluorescent Protein AAC 47 (2003) 309, the paper on which our assay was based), we take the reviewer's point that a reader may incorrectly ascribe the inhibition to some other aspect of the lytic cycle (*e.g.,* intracellular replication), rather than a myosin-dependent motility-based process. We have therefore now: (a) more clearly defined the growth assay as one that measures parasite expansion in culture (lines 132-138); (b) described the myosin-dependent and -independent steps of the lytic cycle (lines 137-140); and (c) added a new figure (new Suppl. Figure 2, lines 140-143) showing that the compound has no effect on intracellular replication.

9) The authors should clarify for the reader (i) why they use in some case myofibrils and other muscle F actin when measuring the Myosin ATPase activity, (ii) what does mean XX% calcium

activation and (iii) why using 75% in these assays which is 3 times higher from the original assays. (iv) Why they did not include non muscle actins in their study since Myosins also extensively work on non muscle actins.

(i) For both striated and smooth muscle myosins, the assays used here are well established and have identified compounds that have translated into animal models of disease. To assay the activity of myosins from striated muscle types, particularly to determine compound selectivity, myofibril assays are preferred as they recapitulate more of the biology as a more "native", membrane-free preparation and respond cooperatively to calcium activation. For cardiac, fast and slow skeletal muscle it is possible to derive high quality myofibril preparations that can be activated by calcium. A reference describing the value of using myofibrils in assays of striated muscle myosin ATPase activity has been added (ref. 71, line 517).

Smooth muscle, a non-striated tissue, is regulated differently and calcium exerts an effect not through binding to troponin as in the striated muscle but through g-protein signaling, with phosphorylation as an end result, making the contraction slower and also much slower to reverse - in line with the physiological role of the muscle. The only way to reliably reconstitute smooth muscle ATPase activity has been through purification and reconstitution of a more reductionist system. The SMM S1 needs to be crosslinked to the actin to achieve high enough local concentrations to generate robust ATPase activity. A reference describing the use of this assay to identify small molecule inhibitors of SMM is now included (ref. 73, line 522).

(ii, iii) Striated muscle myofibrils are responsive to calcium, as muscle contraction is mediated *in vivo* through calcium release from the sarcoplasmic reticulum. Titrating calcium can activate the myofibril ATPase activity up to the plateau (100%) and provide optimal signal to noise and sensitivity for the particular activity being assayed. For counterscreening to determine selectivity, we adjusted the assay conditions to a high basal ATPase activity (75% calcium) to provide high sensitivity for detecting inhibition. A sentence explaining this rationale has been added on lines 519-520.

(iv) We used skeletal muscle actin in all of our in vitro assays since we have shown skeletal muscle actin to be a good substrate for TgMyoA (ref 33, cited on line 536) and skeletal muscle actin can be purified in larger quantities than native actin from parasites or functional recombinant protein from insect cells. Others have also shown that the closely related MyoA from *P. falciparum* moves skeletal muscle actin at the same speeds as recombinant *P. falciparum* actin (Bookwalter et al [2017] JBC 292:19290).

10) The protocol of image analysis of the 3D motility assay was increased to 80 seconds for the test of KNX-002 selectivity using wild type and mutant parasites (Fig 7) when compared to the test of KXN-002 concentration effect on wild type tachyzoites (60 sec in the result section, in Fig 4 legend and in the Methods' section). Is there any specific reason?

> The data for Figure 4 were captured earlier in the project than those of Figure 7 and Suppl. Figure 7. In the intervening time we upgraded our Nikon Elements software from v.3.20 to v.5.11 (as already described on lines 583 and 588). With the upgrade to v.5.11, we also began using Nikon's Illumination Sequence (IS) module, a graphical user interface that provides greater time resolution through a more efficient approach to building the z-stacks and saving the data. With the addition of v.5.11 and the IS module we were able to capture twice the number of image volumes in 80 sec than we were in 60 sec using v.3.20, and that became our standard operating procedure. Other than the improved time resolution, the 60s and 80s assays give indistinguishable relative results. We have now clarified in the methods (line 588-589) that we used the IS module to acquire the data in Figure 7 and Suppl. Figure 7.

11) In the mouse infection experimental design (Method section), it seems that they were no biological replicates in the case of the drug-treated (parasites + mice) which is not the case for the comparison of virulence between MyoA wild type and T130 mutants. If true, and considering what the authors wish to emphasize as a main message, it is fairly complicated to convincingly conclude about the KNX-002 effectiveness in vivo. Maybe the authors could explain their limitations.

> Since we did not know how the compound and parasites would interact in mice – and in keeping with animal welfare standards – we decided that rather than doing multiple replicates with smaller numbers of infected mice we would do a single experiment with a large enough number of mice per treatment condition to ensure that if any animals died unexpectedly or had to be euthanized prematurely we would still have sufficient numbers for robust statistical comparison. Single experiments with ten treated and ten untreated mice are a generally accepted approach in early studies of drug effectiveness (e.g., Ferrreira et al Parasite 2002, 9:261; Rutaganira et al, J. Med. Chem. 2017, 60: 9976; Zhang et al IJP Drugs and Drug Resistance 2019, 9:27), and power analysis shows that if mortality is 100% in untreated mice and 50% in treated mice, 10 mice per group will provide an 80% probability of detecting the difference with a p value < 0.05.

We are also not sure why the compound has been injected only twice, at the time of parasite injection and two days after whereas the mice succumbed after 8 to 9 days even without MyoA inhibitors. Although quite difficult to measure, do the authors have any knowledge (based on the chemistry for example) of the compound stability and lipophilicity in blood and tissues? Because the IC50 on free tachyzoites appears significantly higher (5.3 uM, Fig4) than the in vitro molecular assay, when assessed in motility tests, and is increased for intracellular growth (Fig 8), it is somehow expected that the current compound would not work that great in vivo. Did the author try to provide the inhibitor intravenously every day?

> IP injection is a standard method of administration for early drug treatment studies, and two considerations contributed to our decision to inject on days 0 and 2 post-infection: (a) the

preliminary dose-tolerance studies, which were done with two IP doses of compound two days apart, showed evidence of mild toxicity so we were hesitant to inject more frequently, inject IV, or use more compound/injection; (ii) we expect the compound to work primarily on egressing and extracellular parasites, and since the parasite's lytic cycle takes approximately 48 hours, this two-day injection schedule was chosen to maximize exposure of the extracellular parasites to freshly injected compound early in establishment of the infection. This rationale has now been added to the Methods section (lines 715-721).

In terms of the doing systematic studies of dosing, stability, PK/PD, drug partitioning etc., it is important to restate that the primary goal of this work was to test whether inhibiting TgMyoA activity *in vivo* alters the course of infection. The data reported in the manuscript demonstrated this to be the case. As we state on lines 454-457, "While KNX-002 provided the means to rigorously test the druggability of TgMyoA, it caused weight loss and histological evidence of liver damage in the treated infected mice. Before further animal work, it will therefore be necessary to develop more potent and less toxic analogs that retain specificity for parasite myosin." Our colleagues at Kainomyx have in fact initiated a drug development campaign based on the KNX-002 scaffold and have already identified a derivative named KNX-115, that is 20-fold more potent against recombinant *P. falciparum* MyoA (described on lines 356-361). Given Kainomyx's ongoing efforts we do not believe it makes sense to do any further animal experiments at this time with KNX-002. It will be more informative and ethical to undertake, *e.g.,* dosing, PK and PD studies with the more potent and less toxic derivatives that emerge from the Kainomyx drug development program, once these compounds become publicly available. This does not diminish the importance of the proof-of-principle experiments reported here, which as the reviewer stated, "provide a strong rationale for developing new therapeutic strategies based on targeting MyoA"; rather, it makes it hard to justify doing additional animal studies with a compound that we know will soon be replaced with more potent and less toxic derivatives.

12) Figure 4: 2D and 3D Motility- the authors should comment on the fact that in 2D conditions with 10 uM of KNX-002, circular trajectories (one complete circle so at least 2 parasite lengths but sometimes more) largely dominate over others, whereas in absence of KNX-002 these circular trajectories are barely detectable and helical trajectories predominate. What could that mean as regard to the MyoA functional contribution to either process?

> This is an interesting question that we cannot currently answer. Perhaps helical 2D gliding requires more myosin-generated force than circular 2D gliding, but this is pure speculation at this point. Whatever the explanation, the observation is striking and we believe should be reported as it shows a clear effect of the compound on motility in the widely-used 2D trail deposition assay.

13) Figure 7: Besides the major point raised above for panel C, the information carried by the Figure could be stronger if an additional panel is introduced regarding the interesting assay on the preserved structural stability of the MyoA mutant over the WT MyoA (currently in SupFig7)

> Former Suppl. Figure 7 (now Suppl. Figure 9) addresses one particular explanation for the differential effects of the mutation in the *in vitro* motility assay (Figure 6) and the parasite 3D motility assay (Figure 7). The data in Suppl. Figures 14A and 14B address two other possibilities. For consistency with the other figures and clarity of the narrative, we would prefer to leave the data in Suppl. Figure 9 as a supplemental figure.

14) Material and Methods - Parasite motility assays: remove the duplicated [16] reference.

> Done.

15) The discussion starts with the ongoing debate on mechanisms underlying zoite motility; We found that the work of Pavlou et al. (ACS nano, 2020) should be part of the references listed there, as it brings evidence that a specific traction polar force is required probably in concert with microtubule storage energy at the focal point, a result that questions the prevailing model.

> This was another oversight; the citation has been added (line 307).

16) Concerning the C3-20 and C3-21 compounds, the sentence "they have no effect on the activity of the recombinant TgMyoA (AK and GEW, unpublished data)" in the paragraph starting by "There have been only two previously..." should be refrained unless showing the results.

> We have removed reference to this unpublished work, as suggested (lines 338-340).

17) If possible, the authors should expand more on the effect of KNX-002 on Plasmodium falciparum and its homolog PfMyoA.

> We have expanded our discussion of these preprint data from others on lines 356-361.

Reviewer #3

1) The T130A IC50 was done on the mutagenized clone 5. The authors currently don't have data showing IC50 on the independently generated T130A mutant, to see if the IC50s are similar to one another, or if there were additional resistance mutations present in clone 5.

> Because we did not insert the T130A mutation into a fluorescent parasite background, we cannot directly compare its IC_{50} in the fluorescence-based growth assay to that of the line generated by chemical mutagenesis. Plaque assays do not require fluorescent parasites but, in our hands, these assays lack the sensitivity to reproducibly detect the expected subtle (< 3-fold) change in IC_{50}. While we agree that it would be interesting to know if the mutant generated by chemical mutagenesis contains any additional resistance-conferring mutations, not having this information does not alter the conclusion that the T130A mutation alone reduces the sensitivity

of the motor to KNX-002 (Figures 6-9). See also response to Reviewer 1, comment 1; a discussion of the value of determining what other resistance mechanisms are available to the parasite for this class of compounds is now included on lines 416-422.

2) For 3D motility assays, it is currently unclear from the data and text what the expected maximal inhibition of motility would be; e.g., would parasites depleted of MyoA display 0% motility. Understanding the dynamic range of this assay could help clarify whether this residual 5% motility explains why parasites treated with 20 uM KNX-002 can still form small plaques. This could be achieved by referencing previous work that assesses 3D motility after depletion of a critical motility factor.

> A small fraction of TgMyoA knockout parasites are still capable of motility in 3D (13% when normalized to wildtype for displacements > 2 µm [ref. 46]), so the dynamic range of the 3D assay for TgMyoA-deficient parasites compared to wild-type parasites is 0.13-1.0. The 13% residual motility of the TgMyoA parasites is now referred to on lines 419-420. Treatment of wild-type parasites with 20µM KNX-002 results in a fractional motility of ~0.24 compared to untreated controls (Figures 4 and 7). This less than complete inhibition compared to the knockout is not surprising, since motor activity is not completely inhibited at 20 µM compound (Figures 1 and 6) and parasite growth as assayed either by the fluorescence-based method (Figure 2A) or plaquing (Figure 8) shows greater inhibition at 40 and 80 µM compound than at 20 µM.

3) It would be informative for the authors to discuss the rationale for the selected treatment regime. Since many drug-treatments involve daily dosing, was the two-dose regime based on poor tolerance of the compound in mice or other considerations?

> See response to reviewer 2, comment 11; the rationale for this dosing regimen has now been added to the Methods (line 715-721).

4) Track length is not considered as a parameter in the filament sliding assays (Fig. 6) or the 3D motility assays (Fig. 7). These may be valuable parameters for the authors to examine; however, the time frames analyzed might be insufficient to capture track lengths. Could the authors include analyses of track lengths or discuss the technical limitations of their assays?

> In the *in vitro* motility assays, almost all of the actin filaments move for the entire 60s of video recording so trajectory length is directly proportional to speed and therefore does not provide any additional information. For the parasite 3D motility assay, we have added a new figure (Suppl. Figure 12) showing the effect of the compound on the displacement of wild-type and T130A parasites, along with new text describing these data (lines 269-273).

5) When discussing the minor discrepancies between the results with recombinant protein and parasite motility, the authors could consider the relative concentration of motors in the pellicle;

i.e. it might be necessary to inhibit a greater % of all the motors to truly block motility, perhaps consistent with the higher compound concentrations needed to affect parasite motility.

> This possible explanation has been added to the Discussion (lines 403-406).

6) The authors should include the IC50 data for all 5 KNX-002 resistant clones in the supplementary data. While the 5/26 clones showed >2.5-fold increase in IC50 for KNX-002, it's unclear how the IC50 of the single clone harboring the T130A MyoA mutation compared to the other resistant clones.

> A figure has been added showing these data (new Suppl. Figure 8).

7) For plaque assays, the authors should indicate how much DMSO was used for 0 KNX-002 conditions. It should presumably be the corresponding concentration at 80 µM drug and if not, that control should be performed to account for effects of DMSO at higher concentrations at all drug concentrations tested.

> In all experiments involving treatment with compound, the compound was serially diluted in DMSO to the appropriate range of concentrations prior to dissolving it in aqueous buffer for the experiment itself, enabling an equivalent amount of DMSO to be added to all samples in that experiment, including the DMSO only vehicle controls. This clarifying statement and the final range of DMSO concentrations in each of the different types of experiments has been added to the Methods section (lines 486-491).

8) Authors should indicate the origins of their hexokinase for counter screens.

> The hexokinase used was from Millipore Sigma (#H6380); the supplier has been added on line 507.

9) Authors should indicate µM on graphs.

> The µM label has been added to the graphs where it was missing (Figures 2 and 5, Suppl. Figures 4, 6, 8).

10) In Figures 2A, 5A, and 5B, the use of colored lines (e.g., of different hues) could make the graphs more legible.

> We have experimented with color as a way to discriminate between the different doses on these graphs, but found the use of 8 different colors to be more distracting than helpful. The color-coding approach would be even less useful for readers who have color vision deficiency (including one of our authors). Symbol groupings have been added to the right of all growth

curves to improve the legibility of the graphs.

11) In Figure 2C, it isn't clear which cell line was treated with sodium azide to generate the positive control.

> It was the HFF cells that were treated with azide as a positive control; Figure 2C has been modified to make this clear.

12) In the discussion, "a" is missing in the phrase "...mutation is likely to have long range structural impact..."

> Done.

13) The abbreviation of species (spp.) should be followed by a period.

> Done.

3. Other

Further SAR analyses using an optimized actin-dependent myosin ATPase assay resulted in minor changes to Suppl. Figure 3 and Figure 3, with no significant changes to the conclusions. The text has been modified accordingly (lines 155-161, 178-180).

All other changes to the manuscript not noted above were editorial in nature, made to either improve clarity or correct minor errors in the previous version.
