# Supplementary material for: MyosinA is a druggable target in the widespread protozoan parasite Toxoplasma gondii
Source: PLoS Biol. 2023 May 8;21(5):e3002110. doi: 10.1371/journal.pbio.3002110 (PMC10185354; doi:10.1371/journal.pbio.3002110)
Supplement: Attachment — Submitted filename: Response to editorial comments 230327.pdf [file pbio.3002110.s043.pdf]

Dear Editors:

We have revised manuscript (PBIOLOGY-D-23-00430R1) - [EMID:e75e4c6955318025] to address all comments in your editorial decision letter of March 7, 2023. We have uploaded the revised manuscript as well as five revised figures and 25 new raw data files. The specific changes made are summarized below:

1) "We ask that all individual quantitative observations that underlie the data summarized in the figures and results of your paper be made available Please ensure that you provide the individual numerical values that underlie the summary data displayed in the following figure panels as they are essential for readers to assess your analysis and to reproduce it: Figures 1A, 2ABCD, 4C, 5AB, 6AB, 7BCD, 8B, 9, and Supplementary Figures SF1, SF2, SF3, SF4AB, SF5, SF6AB, SF7AB, SF8AB, SF9AB, SF11BC, SF12, SF13, SF14B."

> All of the required Excel or TIFF raw data files have been uploaded as Supporting Information. The figure legends in the manuscript have been revised to include pointers to the data files, and legends for the data files and other Supporting Information have been inserted into the manuscript following the reference list. The following Data Statement has been entered into the submission system: "All relevant data are within the paper and its Supporting Information files."

2) "We require the original, uncropped and minimally adjusted images … for Supplementary Figures SF10C, SF14A."

> These original figures are now included (S16_Data and S_24 Data).

3) "Please add size bars to the microscopy pictures in Figures 8A and SF11A."

> Done

4) Academic Editor: "I suggest the authors to make less use of emotional enhancers like dramatically, marked, clearly. Just stick to describing what was found and let the reader weigh the evidence herself…. The authors use the phrase 'marked' contrast frequently, at times when the differences are more moderate, e.g. in the mouse experiments where its's used twice. I suggest to go through the manuscript and to remove some of these enhancers and simply state what that there is difference and quantify that difference."

> Done; see changes on lines 332-333, 621, 665, 677, and 686 of the version of the manuscript with changes tracked.

5) Academic Editor: "The title … I feel the word druggable is fine - that is indeed what the bulk of the paper shows - however I would remove the mention of animals in the title. This is a great paper showing that MyoA can be inhibited and it provides new chemical matter to do so, resistance mutants, a bunch of biochemical and cell biological assays - all nicely done and rigorous. The mouse data is a nice add on at the end, but also the weakest part of the study. It's a single experiment that used infrequent dosing due to toxicity and the in vivo potency is not great … most of the mice still die. On its own this experiment does not say so much about the prospect of MyoA as a target. The reviewers offered two avenues: provide more mouse data and show that this is a good drug in vivo (which is difficult and not a bar that the authors should be held to for acceptance here) or t take it out of the title and tone down the abstract a bit (the discussion actually does an entirely fair job at discussing in vivo caveats and the need for further SAR and compound optimization)."

> The title has been changed as suggested, retaining the word "druggable" but removing reference to the *in vivo* results, and the abstract (lines 46-47, 56, and 59 of the version of the manuscript with changes tracked) and blurb have been "toned down" accordingly.

6) Academic editor: "cut "the" in front of cilia (line 536) and "a" in front of few (line 677)"

> Done

<u>Other changes made in revision:</u>

1) Suppl Fig 11 has been revised to include previously missing letters in a few of the compound labels.

2) For consistency with the other figures, the values corresponding to each of the individual biological replicates are now plotted (circular symbols) in Fig 8 and Suppl Fig 11, in addition to the mean values.

3) When assembling the raw data files underlying Fig 4C for upload, we discovered that the time series data for one biological replicate of parasites treated with 5 and 10 µM KNX-002 was incomplete. We have therefore removed these data from our analysis, which slightly changed the data plotted in Fig 4C and the calculated IC_{50}; the conclusions of the experiment remain unchanged.

4) The flow cytometry facility is now more fully acknowledged in the Acknowledgements section, as per their reporting requirements.

5) Any other minor changes made to the manuscript not noted above (but tracked) were editorial in nature, made to either improve clarity or correct minor errors in the previous version.
